# Stigma towards people with a diagnosis of severe mental disorder in primary healthcare centers: perspectives of service users and health teams in Chile

**DOI:** 10.1186/s13033-020-0340-5

**Published:** 2020-02-07

**Authors:** Pamela Vaccari, Raúl Ramírez-Vielma, Sandra Saldivia, Félix Cova, Alexis Vielma-Aguilera, Víctor Victoriano, Natalia Ulloa-Vidal, Pamela Grandón

**Affiliations:** 1 Departamento de Psicología, Universidad de Concepción, Concepción, Chile; 2 Departamento de Psiquiatría y Salud Mental, Universidad de Concepción, Concepción, Chile; 3 Agrupación ex usuarios Salud Mental, Talcahuano, Chile

**Keywords:** Stigma, Primary healthcare, Severe mental disorder

## Abstract

**Background:**

Stigma towards people diagnosed with a severe mental disorder (SMD) is one of the main obstacles for these service users to receive timely and relevant healthcare. This study was undertaken to understand how stigmatizing attitudes are demonstrated towards people with SMD in primary healthcare centers (PHC) from the perspective of those affected and primary healthcare professionals.

**Methods:**

We used a qualitative exploratory research design to contrast the differences and similarities regarding stigmatizing attitudes towards people with SMD in primary healthcare centers (PHC) from the perspective of two groups: (i) people diagnosed with a severe mental disorder, and (ii) healthcare professionals. Data was collected through semi-structured interviews and discussion groups and subsequently analyzed using Atlas.ti software.

**Results:**

Our results indicate that both service users and healthcare professionals manifest stereotypes, prejudices, and discriminatory behavior in health care. In addition, structural aspects of the health system and organizational culture appear to contribute to stigmatization. Both groups agreed that there is a need for healthcare professionals to have more education, specialization, and skill development related to mental health issues.

**Conclusions:**

Interventions to reduce the stigma towards people with SMD in PHC must consider delivery of information about mental disorders, development of skills in the healthcare professionals, and modifications in the culture of the health centers.

## Introduction

Stigma is a relational and social process in which negative characteristics are attributed to groups or individuals based on prejudice that leads to discriminatory behavior [1, 2]. One group that is most affected by stigma is people diagnosed with a mental disorder; in particular, those with a diagnosis of severe mental disorder (SMD). It has been found that healthcare professionals stigmatize service users with SMD [3], and this has important consequences for their healthcare because it creates a barrier to access health services [4], hinders adherence to treatment [5], reduces the quality of medical attention, and ultimately, contributes to a high morbidity and mortality rate in this population [6, 7]. Therefore, reducing stigma towards people diagnosed with SMD is one of the central challenges in the field of mental health [8–10].

Stigma manifested by personnel from primary healthcare centers (PHC) is expressed in different ways and influences the diagnosis and treatment of people with SMD [11]. For example, it has been found that physicians erroneously attribute physical symptoms to the mental disorder which results in physical health problems being minimized or undetected [9, 12]. Furthermore, it was found that physicians have pessimistic attitudes about the recovery of people with SMD [13, 14]. For instance, a study found that the physician’s perception of the patient’s adherence to treatment was influenced by stigma, in other words, low adherence was attributed to the psychiatric diagnosis [15]. In addition, PHC professionals believe that this population should be attended by specialists, so they tend to have avoidance attitudes towards them. In general, healthcare professionals have more negative attitudes towards people diagnosed with SMD than towards service users with other psychiatric diagnoses [16]. Moreover, stigma towards people with SMD in healthcare professionals is related to negative experiences with service users, a perception of lacking the skills to manage this population, and a shortage of information and training in mental health [13, 17].

For some years now, stigma has been considered an ecological phenomenon that includes structural, interpersonal, and intrapersonal variables and, therefore, the form it acquires depends on the context where it is expressed [18]. Thus, understanding how stigma is manifested in different contexts would aid in the development of culturally relevant interventions to reduce it [3]. For example, when stigma occurs in the workplace, it is recommended to have interventions that consider the different variables present in the work environment [19, 20]. Several studies highlight the importance of understanding the form that stigma takes in the healthcare system, i.e. taking into account both the individual and social aspects before developing an intervention to reduce it [21, 22].

In Latin America, there is little information about how stigma occurs in different populations, especially in healthcare professionals [23]. In a recent systematic review on the subject in South America, it was found that there were some differences in how public and family stigma was expressed in contrast to Western European countries [24]. For example, it was determined that there were more attitudes of compassion and benevolence associated with the Latin American culture due to the social capital of these groups. Hence, the conclusion was that interventions must include the cultural dynamics of the Latin American population [24].

This study is part of a larger investigation aimed at developing an intervention program to reduce stigma in primary healthcare professionals in Chile called “Equal-Mind”. In Chile, PHC serve the majority of the population and operate based on a model of family and community health. The two main family health agencies are the Family Health Centers [Centro de Salud Familiar (CESFAM)] and the Family Health Community Centers [Centro Comunitario de Salud Familiar (CECOF)] that are made up of multidisciplinary teams that provide healthcare services for the population within a given territory [25]. The objective of this study was to determine how stigmatization occurs towards people diagnosed with SMD in PHC, based on the experiences of those affected and PHC healthcare teams. Our results will help in the design of a contextualized intervention to reduce stigma in PHC professionals.

## Materials and methods

### Design

We used a qualitative exploratory design [26, 27] that included: a pre-exploratory phase; a methodology defining phase for the discussion groups and semi-structured interviews for data collection; contact with gatekeepers; field work; data analysis; and triangulation.

### Participants

The sample consisted of people with a diagnosis of SMD and primary healthcare professionals. The inclusion criteria for people diagnosed with SMD were: receiving healthcare in PHC, having a diagnosis of SMD for at least 1 year, being over 18 years of age with autonomy to sign a consent form, and agreeing to participate in a group discussion or interview. The inclusion criteria for healthcare professionals were: working for more than 1 year in a PHC and agreeing to participate in group discussions. Healthcare personnel who only had administrative tasks were not included in this study.

The service users that participated were recruited from associations formed by people having a diagnosis of SMD. They were referred to these associations by mental healthcare professionals based on their diagnosis.

The discussion groups and interviews were held in the communities of Talcahuano and Concepcion between April and October 2017.

### Procedure

#### Pre-exploratory phase

Recent research was reviewed on the perception of stigma in healthcare professionals towards people diagnosed with SMD. These studies provided relevant themes for the interview and group topics to be discussed with healthcare professionals and service users.

#### Methodology defining phase

We created a topic guide in order to define the conversation for the discussion groups and interviews. The informed consent was also prepared in this phase.

#### Gatekeepers contact phase

We contacted the leaders of service users and family group associations, directors of PHC, and professionals from the City Council Disability Office. The selection of communities in this study was based on convenience. These groups of people provided us with information about potential participants for this study, who were then contacted by the research team.

#### Field work phase

Data was collected through discussion groups and semi-structured interviews.

Four discussion groups were held: two groups with people diagnosed with SMD, each one with 12 participants; and two groups with healthcare professionals with 6 and 7 people respectively. The group discussions lasted between 60 and 90 min. In order to obtain the maximum sample variability for the service user group, we combined the dimensions of “community” (Concepcion-Talcahuano) and “sex” as a heterogeneity criterion, and the diagnosis of SMD as a homogenization criterion. With regards to healthcare professionals, the dimensions of “Health Center” (CECOF-CESFAM) and “sex” were used as a heterogeneity criterion, and “professionals” as a homogenization criterion. According to these criteria, the total number of subjects participating in the discussion groups was 37 (Table 1).

**Table 1:** Characteristics of focus group participants

	SMD^a^ user Concepción	SMD user Talcahuano	Professional CECOF^b^	Professional CESFAM^c^
SMD user Male	5	6		
SMD user Female	7	6		
Professional Male			4	1
Professional Female			4	4

^a^Severe Mental Disorder

^b^Family Health Community Centers

^c^Family Health Centers

In the case of healthcare professionals, only discussion groups were held due to difficulty in meeting with them and their lack of time for interviews. The high demand for attention in the public health system in Chile gives very little room for healthcare providers to carry out other activities. This situation also affected the number of professionals who participated in the discussion groups.

Five people diagnosed with SMD were selected for the interviews using the snowball sampling technique, with the gatekeepers’s help [27]. The interviews lasted between 40 and 60 min. The sample was reduced due to difficulties in accessing people who met the inclusion criteria, which were: having a diagnosis of SMD for at least 1 year, being over 18 years of age with autonomy to sign a consent and no participation in discussion group. Within the user’s associations, people who met the inclusion criteria had already participated in the discussion groups and, therefore, a small number was available for interviews (Table 2).

**Table 2:** Participants who met the inclusion criteria for semi-structured interviews

	SMD^a^ users Concepción	SMD users Talcahuano
SMD user Male	0	2
SMD user Female	0	3

^a^Severe mental disorder

The general criterion of this qualitative sample coincided with the criteria of convenience and heterogeneity [28]. In addition, to disclose the magnitude of variation and differentiation in the population, caution was exercised when accessing the most typical and atypical cases [27, 28].

#### Data analysis phase

Data was analyzed using the thematic analysis technique. The individual interviews and discussion groups were analyzed merged on the whole. We started by familiarizing with the data by reading and re-reading the text several times, developing some ideas about the nature of the data and its relevance in relation to the research question. Later we coded the data through the text line-by-line, in order to identify meaning units, and labelled them with a code that captures the meaning identified. The choice of codes was made by inductive and deductive approach to observe emerging concepts that could be more contextual and pertinent to the particular experience of the participants. A thematic grid was created to organize the results [27]. We used the qualitative data software Atlas.ti 7 (Scientific Software Development GmbH). Each type of data was initially analyzed separately and then merged until the code and category were created. Data analyses were done taking into account some theoretical concepts obtained from the literature review. Additionally, these issues were considered and included in the final analysis results. The final categories considered were: stereotype, stigma, prejudice, discrimination, self-stigma, and role. In addition, several emerging issues that were viewed as problematic by the participants were also considered.

In both cases, discussion groups and interviews, participants incorporated their feedback through the clarification technique. This means that after transcriptions had been conducted, participants were re-contacted by mail or face to face, and given the opportunity to check if they needed to make any clarification on what was written.

#### The triangulation phase

Researcher’s triangulation was made in order to analyze the data. This process was made by three researchers separately, then the analyses were shared and compared, and the final codes were obtained. The data was coded up to the saturation point, i.e. when no new categories could be identified, and until new instances of variation for existing categories ceased to emerge, according to the research team.

In order to ensure the reliability of this study [28], we used the criteria of credibility, transferability, and dependability. *Credibility* was done by the triangulation analysis between researchers; thematic analyses were done by three researchers separately and then compared. Regarding *transferability*, caution was exercised through the adequate use of qualitative sampling that considered a selection of relevant contexts by type, variation, heterogeneity, and convenience; and, also, by including selection filters for inclusion and exclusion of participants. Finally, the *dependability or reproducibility* of this study was determined by the revision and audit by another group of experts external to this research team, as well as by observations from the Ethics Committees from University of Concepcion and from the Health Services of Concepcion and Talcahuano.

### Ethical aspects

The study was approved by the Ethics Committee of the University of Concepcion and the Ethics Committees from the Health Services of Concepcion, code CEC 16-08-44, and Talcahuano, code 67. All research procedures described up to that point were approved. Protocols were designed taking into consideration the rights of the participants included in the Declaration of Helsinki. All participants voluntarily agreed to take part in the study by signing an informed consent that protected the confidentiality for later use of the information.

## Results

The topics for people diagnosed with SMD were:

- Attitudes and behavior of healthcare professionals (prejudice, stereotypes, stigma, discriminatory behavior)
- Strategies used by service users to improve their medical attention
- Bureaucracy of the health system (waiting, asking for doctor appointments, referrals, etc.)
- Expectation of the relationship with health professionals (expected role)
- Self-stigma on the part of people diagnosed with SMD
- Ethical-professional training in mental health (expected role).

The topics for healthcare professionals were:

- Institutional culture of treating people diagnosed with SMD in PHC
- Lack of knowledge about mental health
- Stereotype and stigma towards people diagnosed with SMD (label, negative prejudice)
- Professional role
- Health system resources.

The categories and their respective arguments are presented below.

### Topics for people diagnosed with SMD

#### a) Attitudes and behavior of healthcare professionals (prejudice, stereotypes, stigma, discriminatory behavior)

This topic involved behavioral and attitudinal aspects of healthcare professionals with regards to the way they treat service users with SMD, e.g. reprimanding them when attending them, avoiding them, exhibiting them to a clinical round with students without their prior authorization, not attending them if they come alone, and rejecting them as a result of their appearance. Because of this treatment, service users expressed feelings of being disqualified and disrespected, which demonstrates that they understand their rights and can perceive when these rights are violated. Furthermore, they repeat the terminology that healthcare professionals use when referring to them which contributes to the process of self-stigmatization; for example, they call themselves “mentally ill”. Finally, there is a kind of positive discrimination that is demonstrated by faster medical attention in order to reduce the time assisting them and to prevent them from becoming agitated.

*“I don’t know, but they always tell me “You’re here again!”, they say. The paramedics, another paramedic said to me “Why are you here again? You were just here not too long ago…”* (Reprimand, avoidance of contact).

*“Students came in, so I was there showing my teeth, showing everything. What a drag. Even in psychiatry the same thing happens to me, I already told the doctor, the doctor already knows, I’ve been interviewed like four times, I feel so embarrassed, but I don’t like that, so I decide that when the students come in…I’ll participate”* (Being exhibited during a clinical round with medical students without authorization, annoyance, focus on rights).

*“This young man here, the little girl here too; see the appearance, see the appearance… for example of any… of any defect that the person has, with respect to … either more chronic or more severe… they think they can’t fend for themselves, like handicapped people… they discriminate based on appearance”* (Reference to discrimination by appearance).

*“If I go by myself, they don’t pay attention to me because they say that a person like me can’t show up alone to receive medical attention. They say that a responsible adult has to be with me”* (Refused attention because they come alone, discrimination, avoidance).

*“This may sound quite frank, it goes straight to the bone as some would say, but the truth is… they don’t pay attention to us, they don’t see us as people, only numbers. Even if you present yourself as you are…that you have problems talking, they criticize you”* (Discrimination, prejudice)

*“Doctors… who else… dentists, the attention was good. Since they knew the problem, they treated me like more… quicker, let’s say…maybe because they thought I was going to hit them, that I was going to go crazy and that I would certainly hit them (laughs). That’s why they had respect for me”* (Decrease in attention time due to fear).

#### b) Strategies used by service users to improve their medical attention

These are the methods that service users have developed in order to receive better health care. They perceive that healthcare professionals tend to negatively discriminate against them, not attend them at all, or avoid them; so, they have found ways to prevent this from happening. Some of the strategies they use are: not to show up alone but to be accompanied by someone, to show the healthcare professionals that they have social skills, and to have a kind demeanor.

*“Once I went with my father. That time the attention was good, but when I go by myself it’s different”* (Going accompanied is a way to receive better attention).

*“I have tried to turn this situation around. I try to be more sociable so that people like me, I try to get along better with them”* (Show social skills).

*“And no, what*’*s more, when I go to… in this moment to… this week I have to go get my medicine, if they treat me good, I take advantage of it and ask for an appointment and they give me one right away: I have no problem coming back tomorrow or another day… Like I say, as a child my mother and father taught me: If you want people to respect you, you must respect them”* (Be kind to receive better attention).

#### c) Bureaucracy of the health system (waiting, asking for doctor appointments, referrals, etc.)

This category refers mainly to the issue of excessive administrative procedures associated with healthcare that usually makes it more difficult to access this health benefit. This situation is common to all service users of the public healthcare system in Chile; however, people diagnosed with SMD experience tension with this situation and often times feel discriminated against.

*“One morning when I went there to get some exams done, I waited a long time but the girl didn*’*t call me. Another woman was called before me so I said to the girl, it’s my turn now, not that lady’s turn, and I left and went home feeling sad”* (Consequences of waiting a long time to be attended).

*“And I remember that we got to the doctor’s office about three in the afternoon. I’m not lying, but we were there until almost nine thirty at night, I mean we spent the whole afternoon waiting for*… *for someone to see my friend, until they finally saw him; so yeah, they saw him like at quarter past nine but we didn’t leave until like nine thirty”* (Waiting to be treated).

#### d) Expectation of the relationship with health professionals (expected role)

In general, people diagnosed with SMD usually have negative expectations about the treatment they will receive in the PHC. Nevertheless, they understand that they have rights as patients and deserve adequate attention, so that is why these situations are frustrating and give them the feeling that they are being discriminated against and stigmatized.

*“So, since they see us differently, because we have an illness, it’s like they brush us off to the side and for me everybody is the same, they have the same rights, mentally ill or normal, it’s like… you make an appointment, you need to be punctual, to have more patience, and calmness to treat that person because he got up early to stand in line, and what’s more, he gets rejected, they ignore him and others go before him; in the end, all CESFAMs are a drag”* (Expectation for good attention focused on respect and patient’s rights not met)

*“Treat patients good. Change your attitude and quit frustrating people”* (Expectation for good attention focused on respect and patient’s rights not met)

#### e) Self-stigma on the part of people diagnosed with SMD

This category refers to prejudice against oneself that occurs when a person with SMD internalizes the stigmatization from the psychiatric diagnosis. They believe their diagnosis is unchangeable and justify their condition with a label, and thus describe themselves within the boundaries of their mental health label. For example, they define themselves as “someone who has a problem” or “mentally ill” or “someone you have to be patient with”, which perpetuates their self-stigma.

*“Ma’am, just to let you know, that I, for example, that every time I go to any medically related place because of health problems, you can see I*’*m schizophrenic, and I have to take my pills, besides other stuff”* (Self-identification because of diagnosis)

*“Of the… those that give me medical attention should be more prepared, to understand what it means to be mentally ill”* (Self-identification as “mentally ill”).

*“In the health center the professionals don*’*t know the patients, they don*’*t know each other well. And for example, it takes a lot of patience for*… *for mental health”* (Person believes that their diagnosis requires “patience”, which indicates a type of self-stigma to think of oneself as someone who needs to be treated with patience).

*“That’s why (referring to the diagnosis) I find it hard to learn, that is what the therapist told me*…*”* (internalizes negative vision of health providers and self-devalue)

#### f) Ethical-professional training in mental health (expected role)

This category refers to the need to have health professionals that are trained in mental health issues, who understand patient’s rights and are respectful toward people regardless of their diagnosis and/or situation in which they find themselves.

*“I think the professionals, like the patients, like the case of this young guy here, uh*…*sometimes*…*the professionals aren*’*t trained or prepared to deal with those kinds of cases; for example, when they think you have a deficiency, they think you can*’*t do things by yourself, that*’*s why they say someone has to be with you. So yeah, I think that…just that*… *Well, a while ago we did a*… *meeting, it was pretty good, we sent letters to the professors in charge of the courses at the universities, certain universities, to the student body, so that the psychologists, social workers, even professors, could… could understand what it*’*s like to have a mental deficit, in our case, to put*…*to put themselves in our shoes, because sometimes, textbook theories don*’*t depict our reality”* (Account that requires professionals to be more sensitive to the topic).

*“As I said (name), you want people that are more qualified on the topic of mental health, mental health. Once we attended a group*… *uh*… *for mental health*… *they need more capable people*… *people who understand*… *what mental health is all about”* (Reference to the need for preparation on mental health issues required by professionals).

### Topics for PHC professionals

#### a) The Institutional culture of treating people diagnosed with SMD in PHC

This category deals with the habits and customs of treating people diagnosed with SMD in CESFAM. There are three types of situations: (1) there is a fear of people with SMD in some PHC due to previous unpleasant experiences. Therefore, there are feelings of vulnerability and avoidance behaviors; (2) there are PHC where the health teams have a designated person, usually someone who has better relational skills, to be in charge of the first encounter with a service user diagnosed with SMD. This is a demonstration of institutional culture that permeates the form of attention provided; (3) there are PHC where the professionals treat people diagnosed with SMD with a more defensive attitude and tend to minimize their health symptoms. These professionals use nicknames and disqualifications towards service users with SMD because they believe that they magnify and exaggerate their symptoms in order to get treated more quickly. These three types of situations occur in every PHC either alone or in combination. Moreover, within these groups there also occurs endo and exo-group phenomena so the form of treatment adopted at the PHC is usually replicated without resistance by other people who share the workspace.

*“About these aggressions, for example, we*’*ve had service users, I remember last year when a secretary didn*’*t give, didn*’*t give a patient an appointment, and he left and slashed the car tires, so there is a feeling of vulnerability”* (Culture of fear and avoidance).

*“The protocol is always like, as a coordinator I have to accept the service user, and one tries to avoid that the patient talks with another one to prevent the situation from escalating, so one tries to somehow have a type of containment*…*”* (Institutional culture of service user acceptance).

*“In the meetings, in the meetings of, for example, in the meetings that I attend because I am a coordinator and have to attend meetings in CESFAM, they always talk about the priority given to the loony patients”* (Culture of minimization of people with SMD by labeling them as “loony patients”)

*“They are always… I don*’*t know if they do it on purpose, I mean they are full of problems, but they take advantage of that problem, they take advantage of it, they take advantage of it because they know that they have a different health problem, true, and so they look for the right professional to decompensate in front of, so they can get what they came for, so in a way they also take advantage of their condition”* (Culture of minimization of the general health conditions of people with SMD).

#### b) Lack of knowledge about mental health

In this category, professionals recognize that they have little knowledge about mental health problems and how to approach them. This ignorance contributes to stigmatization because they have wrong ideas that lead them to generalize about negative experiences that may be due to other causes and not to the diagnosis of SMD.

*“There has never really been a, as you say a workshop to evaluate and differentiate, as she says, what mental health really is…”* (Ignorance about how to approach mental health)

*“All of a sudden he grabbed my arm and*… *it was like a struggle and he started treating me bad, and, I was like ‘oh no, what do I do?’ because quite frankly we are not given any training or preparation”* (Fear associated with ignorance about how to deal with people with SMD).

#### c) Stereotype and stigma towards people diagnosed with SMD (label, negative prejudice)

This refers to the conceptualizations that health professionals have towards people with a diagnosis of SMD. Generally, they are based on a lack of knowledge and information, and they are usually negative, totalitarian, and rigid. For example, their descriptions highlight the negative behavior of people with SMD, and their lack of behavioral and emotional control is attributed exclusively to the diagnosis. They point out that they are disturbed and aggressive people that are dependent and with little capacity to make decisions.

Furthermore, health professionals do not trust what SMD patients say because they interpret their demeanor as a strategy to obtain some sort of benefit and thus neglect to provide them with attention. In addition, there may be little empathy towards an SMD patient because of the belief that they are not normal people. Consequently, these attitudes propagate discriminatory behavior towards people with SMD.

*“When she came in, we all recognized that lady, that she is a very abusive woman towards her husband, but she started carrying on and began acting like the victim, in front of all the service users, and that*’*s when one begins to stigmatize the person, so one says, wow, this lady is really crazy…”* (Strategy towards someone with SMD).

*“What happens is that in a certain way they victimize themselves and they lose control easily, they are quite demanding; so, as they say, they exacerbate everything”* (Referring to the fact that people with SMD tend to change and exaggerate things when they request attention).

*“Even more serious, so I believe it has to do with being more regular and improve certain practices, in other words, we had mental health patients who were taking the same dose of medication for more than a year and they were never evaluated by a psychologist, or we*’*ve had mental health patients who replicated prescriptions without any supervision…”* (Neglecting attention and not supervising the use of medication).

*“And the way they have to be treated, it has to be different and it has to be special. The fact that they are dependent, I mean, some understand better than others, others that don*’*t understand much depending on the damage, the mental cognitive deficit they have; when it*’*s more severe, there*’*s not too much they can, they can understand”* (Description of the negative perception professionals have towards people with SMD)

#### d) Professional role

This refers to understanding the positive effect the professional role has on the service user*’*s experience, as well as understanding the impact of their role when people do not receive adequate treatment. Additionally, it involves empathy and responsibility towards healthcare attention as an important task.

*“And in the end you start talking; well, my experience has been, to take them aside to a quiet area, and talk with them, just start talking, and in the end you realize that they*’*re not crazy, they*’*re just someone going through a rough time, something that could also happen to you at some point*…*”* (Work role that transforms, empathy, and responsibility).

*“We have a great responsibility as professionals because it*’*s not just a service user, an ID number that comes in. Sometimes, they just need someone to listen to them, and what happens, I*’*ve realized, how the human mind is, that is, at this point in life when one has gone through periods of crisis, what do I know?, I really want to feel fortunate and thank God that I have been able to come out of these crises because one encounters many problems in life, and you see another human being and you only see what*’*s happening on the outside, but they don*’*t have the capacity or strength to get better, so then, it could be something simple, at least one sees it as simple, but the person feels like they are in a tunnel*… *“*(Work role that transforms, empathy, and responsibility).

#### e) Health system resources

There is a large demand on the healthcare system and the human resources to cover it are not always available, which leads to frustration for the service users and promotes situations of aggression towards the healthcare professionals. Also, the professionals are aware of the impact that the scarcity of resources has on getting medical appointments and treatment, which produces stress for them. Therefore, a vicious cycle occurs based on the lack of human resources that leads to frustration in both service users and healthcare professionals.

*“There was a time, a long time, when there were few doctor appointments and we were constantly attacked; then, suddenly, of course, there were many people who could put up with it, but there were others who after constantly being exposed to this abuse just*…*cracked”* (Assault due to lack of medical appointments).

## Discussion

This research is one of the first in Latin America to evaluate how stigmatization occurs towards people diagnosed with SMD from the perspective of those affected and PHC professionals. The results demonstrate that both service users with SMD and health professionals perceive discriminatory behavior towards people diagnosed with SMD, similar to that found in other studies [11]. However, the way in which discrimination is experienced is different for both groups. For example, service users perceive discrimination in the form of rejection and abusive behavior during attention, whereas professionals discriminate service users by minimizing their physical symptoms and attributing them to their diagnosis. In addition, there is an attitude of distrust on the part of professionals towards the service user that stems from an ethical judgement, i.e. they are seen as people who exaggerate their symptoms to obtain some benefits. For example, in the case of over-demand in the Chilean health system [29], this would mean being treated more quickly. Based on the view that professionals have towards service users, they have established a vision of what “good service users” are, those that adapt to the system, and what “bad service users” are, those that take advantage of their condition. This moral component of stigma that is related to what is culturally approved of in a particular context has been described in other studies [30, 31], although not specifically in health professionals. Furthermore, health professionals stereotype people diagnosed with SMD as “dangerous” [3, 13], and this serves as a positive discrimination towards these service users because it results in them being attended in a preferential way to avoid any possible risks associated with their behavior [32].

In agreement with other studies, people diagnosed with SMD exhibit self-stigma [33, 34] because they assume that their condition is irreversible based on the view that society has of their clinical diagnosis, and they justify their symptoms based on the psychiatric label given to them.

In addition, service users with SMD use active coping strategies against the anticipation of discrimination. As previous studies have shown, people with SMD display anticipatory stigma, i.e. they perceive they will be discriminated against, although it has not occurred yet [12]. Hence, they try to be accompanied by a direct relative when they go to the PHC and/or use their social skills to please others. These strategies demonstrate the ability of people with SMD to counteract discrimination, which is in agreement with other studies [35, 36].

Anticipatory stigma of the relationship with the health professional is contrary to the rights of patients as stipulated by Chilean legislation [37]. This incoherence between what is expected from the health system and what actually occurs produces tension and frustration in the service users. On the other hand, the professionals understand that the idea of their role as “healers” has a relevant impact on the service user’s life. Therefore, an adequate treatment towards the service user should be a part of their daily task. This concept has been promoted by the family health model in Chile where satisfaction and treatment of service users are key elements that are evaluated in PHC [38]. However, this vision is in conflict with those PHC that are based on fear and marginalization of people with SMD, as demonstrated in our study. This contrast between what sounds good in theory and what happens in practice leaves the professional confused and helpless to modify the situation.

Both service users and health professionals agree that there is a need for more mental health training in PHC professionals. This is an important point because ignorance can lead to negative characteristics being attributed to the behavior of people with SMD resulting in stereotypes that become reality [3, 12]. Therefore, training in mental health is important to acquire skills that contribute to solving difficult situations [21, 39] and knowledge that demystifies erroneous beliefs, thus resulting in better treatment [22, 40]. In addition, ethical-professional training related to the role that is expected of healthcare professionals is also essential. Furthermore, it is necessary to have professionals trained in patient’s rights and who are respectful towards service users regardless of their diagnosis and/or situation [22]. On the other hand, service users should also receive training because people who understand their rights can better handle situations of abuse and stigma; thus, education is important in this regard.

A relevant aspect of our results is the influence that meso and macro social variables have on stigma. For example, the organizational culture becomes very important at the meso level because more constructive cultures predict positive work attitudes that favor better interactions between healthcare professionals and service users with SMD [41]. The macro social level, on the other hand, is marked by economic resources that are reflected in the lack of human resources, especially physicians, and in the bureaucracy of the health system. Unfortunately, there is a deficit of physicians in the Chilean primary healthcare system, despite the implementation of a family health model that has contributed to the training of specialists specifically for PHC [37, 42]. Work overload, unsatisfactory salaries, and the lower social status associated with a doctor who works in a PHC are among the factors that are partly responsible for this deficit [43]. The bureaucracy of the system is manifested mainly by the delay in attention which is a result of the existing procedures. Thus, for service users with SMD, this becomes an important obstacle because when they are mistreated or ignored by professionals, this usually leads to even more postponement of their attention. These results support studies that view stigma from an ecological approach. Therefore, in order to reduce stigma, it is not only necessary to target attitudes, stereotypes, and discriminatory behavior, but also to consider aspects related to the social context of the individuals [18]. Stigma is produced and reproduced within these social contexts and the form it takes depends on the characteristics of the social environment. Therefore, modification of attitudes that are formed in these particular environments must also be done within the same context. For instance, for some years now, there have been interventions called “workplace” that consider work related aspects to help introduce changes in the way work is organized and developed [19, 20].

One of the main limitations of this study was the use of a convenience sample that consisted of people diagnosed with SMD who participated in service user and family groups. As a result of their social participation, this group has a greater awareness of prejudicial attitudes and discriminatory behavior which makes it difficult to extrapolate the results to other people with SMD. Therefore, it is important to repeat this study with service users who do not belong to any social organizations and compare the results. Also, the professionals who participated in this study corresponded to a small number of communities in Concepcion and Talcahuano, Chile. Furthermore, another limitation was that the sample of professionals did not reach the saturation of topics; therefore, future studies should include a greater number of participants. In addition, future studies should examine how the meso and macro social aspects influence stigma from the perspective of the decision makers involved in health management, since they have a better understanding about these facets.

## Conclusions

This study demonstrated that stigma in healthcare professionals from PHC is marked by stereotypes, attitudes, and individual behavior, aspects of the PHC, and the structural context of health in Chile, i.e. micro, meso, and macrosocial work areas. At the individual level, service users with SMD perceive stigma as rejection and discriminatory behavior, and healthcare professionals manifest stigma by discrediting the service user because of the stereotype linked to their diagnosis. Within the health system there exists a vision of ‘good’ and ‘bad’ patients that allude to a cultural construction based on the characteristics of the healthcare system. Therefore, in order to intervene in stigma, it is not only necessary to understand the contextual meaning that it has, but also the social factors that contribute to its expression.

Our results confirm the need to use programs with an ecological approach to reduce stigma that consider different intervention levels. Also, our study demonstrates the need for the public health system in Chile to improve healthcare attention for service users and implement available resources, which are determining factors in stigma towards people diagnosed with SMD.

## Data Availability

The datasets generated during and/or analyzed during the current study are available from the corresponding author on reasonable request.
